# Differential *Plasmodium falciparum* surface antigen expression among children with Malarial Retinopathy

**DOI:** 10.1038/srep18034

**Published:** 2015-12-14

**Authors:** Abdirahman I. Abdi, Symon M Kariuki, Michelle K. Muthui, Cheryl A. Kivisi, Gregory Fegan, Evelyn Gitau, Charles R Newton, Peter C. Bull

**Affiliations:** 1 KEMRI-Wellcome Trust Research Programme, P.O. Box 230-80108, Kilifi, Kenya; 2 Department of Biochemistry and Chemistry, Pwani University, P.O. Box 195-80108, Kilifi, Kenya; 3 Nuffield Department of Clinical Medicine, John Radcliffe Hospital, University of Oxford, Oxford, OX3; 4 Department of Psychiatry, University of Oxford, Oxford, UK; 5 Department of Parasitology, Liverpool School of Tropical Medicine, Pembroke Place, Liverpool, L3 5QA.

## Abstract

Retinopathy provides a window into the underlying pathology of life-threatening malarial coma (“cerebral malaria”), allowing differentiation between 1) coma caused by sequestration of *Plasmodium falciparum*-infected erythrocytes in the brain and 2) coma with other underlying causes. Parasite sequestration in the brain is mediated by PfEMP1; a diverse parasite antigen that is inserted into the surface of infected erythrocytes and adheres to various host receptors. PfEMP1 sub-groups called “DC8” and “DC13” have been proposed to cause brain pathology through interactions with endothelial protein C receptor. To test this we profiled PfEMP1 gene expression in parasites from children with clinically defined cerebral malaria, who either had or did not have accompanying retinopathy. We found no evidence for an elevation of DC8 or DC13 PfEMP1 expression in children with retinopathy. However, the proportional expression of a broad subgroup of PfEMP1 called “group A” was elevated in retinopathy patients suggesting that these variants may play a role in the pathology of cerebral malaria. Interventions targeting group A PfEMP1 may be effective at reducing brain pathology.

In children living in sub-Saharan Africa, severe malaria presents in three overlapping syndromes (severe malaria with impaired consciousness, severe malarial anemia, and severe malaria with respiratory distress)^1^. Severely impaired consciousness or deep coma associated with malaria is referred to as cerebral malaria (CM) and in early studies was shown to be associated with accumulation of parasite-infected erythrocytes (IE) in the cerebral blood vessels^2^,^3^,^4^.

Autopsy studies of fatal malaria show large numbers of erythrocytes containing mature stages of *P. falciparum* adhering to the endothelia of the brain microvasculature^2^,^3^,^4^, obstructing blood flow. In fatal instances of coma in children with malaria parasitaemia, the percentage of capillaries containing sequestered malaria parasites can differentiate children who died from malaria from those who died of non-malarial causes but happened to carry parasites^4^. The challenge has been to find markers of parasite sequestration in the brain in non-fatal cases of severe malaria. Malaria retinopathy has been of great interest in this regard. Malaria retinopathy, characterised by retinal whitening, vessel color changes and retinal hemorrhages^5^,^6^ has been found to be a surrogate marker of parasite sequestration in the brain^4^ and is the most specific clinical indicator of cerebral sequestration^7^,^8^,^6^.

Sequestration occurs as a result of interaction between IE surface ligands and receptors on the endothelial cells that make up the inner wall of the microvasculature (reviewed in^9^). *P. falciparum* erythrocyte membrane protein one (PfEMP1), is a diverse family of parasite proteins that are inserted into the surface of the IE. These molecules play a central role in cytoadhesion of IE to the endothelia of the microcirculation^10^. Each molecule contains a number of functional cytoadhesive domains, with different broad classes of domains having different cytoadhesive functions to various host molecules such as ICAM1, CD36, complement receptor 1^11^ and endothelial protein C receptor^12^. PfEMP1 is encoded by a diverse family of about 60 *var* genes per parasite genome^13^. These genes can be classified based on their upstream 5′ un-translated region (5′ UTR) into different functional groups A, B, C and E^14^. An alternative classification based on combinations of cytoadhesive domains called domain cassettes (DC) has recently been described^15^. Switching between *var* genes modifies the antigenic and binding properties of IEs^16^, and is likely to play a role in the distribution of parasites throughout the body. DC8- and DC13-containing PfEMP1 were recently selected on human endothelial cells^17^,^18^ and transcript levels obtained with primers designed to detect these PfEMP1 subtypes were found associated with severe malaria^19^. These subsets of *var* genes have been proposed to express PfEMP1 with specificity to endothelial protein C receptor (EPCR)^12^. These studies suggested a mechanism for the pathology of cerebral malaria in which binding of parasites to EPCR drives inflammation and endothelial activation^12^,^20^. As malarial retinopathy is considered a surrogate external marker for IE sequestration in the brain, the PfEMP1 subsets associated with strong binding to endothelial cells are expected to play a role in retinopathy. We therefore explored the relationship between the expression of various *var* subsets and malaria retinopathy as a way of dissecting the relationship between parasite sequestration in the brain and disease pathology.

## Results

### Clinical characteristics of patients

The clinical features of the children included in this study were described in Table 1. Of the 140 children in the previous study^21^, who fulfilled the WHO definition of cerebral malaria^22^ and had retinopathy status examined, 80 had available stored RNA samples for *var* expression analysis. Retinopathy was positive (CM-R^+^) in 25/80(31.25%), and negative (CM-R^–^) in 55/80 (68.75%), which is similar to the original study^21^, suggesting good representation of the original sample.

For five of the samples, PCR amplification of the reference genes could not be achieved and therefore samples from 75 children were analyzed for *var* expression, of which 52 (69.33%) were CM-R^–^ and 23 (30.66%) were CM-R^+^. Table 1 shows the clinical characteristics of these 75 children. Children with coma co-presenting with respiratory distress (RD) tended to be more common among the CM-R^-^ group, though this was not statistically significant (p = 0.08, Table 1). Consistent with previous studies^23^,^24^,^25^ the CM-R^+^ children tended to be more anemic with lower hematocrit, hemoglobin and erythrocyte counts (hct; z = 3.0 p = 003, hb; z = 3.0 p = 003, RBC count; z = 3.4, p = 0.0006, Table 1). Elevated plasma PfHRP2 concentration was also associated with the CM-R^+^ group (z =  −2.2, p = 0.03 Mann-Whitney U test, Table 1) as observed previously^25^. However, for two markers of endothelial activation (angiopoietin-2 and soluble ICAM-1) previously associated with retinopathy^24^ the difference between the two groups was not significant (Table 1).

### Retinopathy is associated with a higher proportion of group A transcript but lower overall *var* transcript quantity

We compared the expression of *var genes* in parasites isolated from children clinically diagnosed with CM-R^**+**^ and CM-R^**-**^ by quantifying the expression of various *var gene* subsets in parasites from the two groups of children using the primers listed in Table S1.

**Figure 1:**
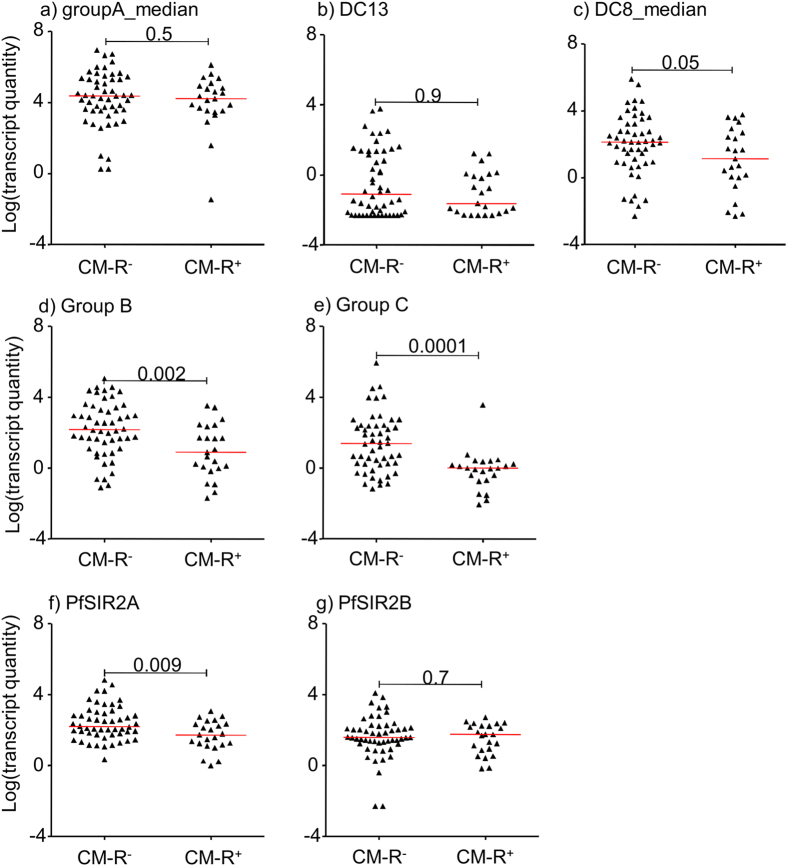
*var* transcript quantity and retinopathy. Dot plots showing the transcript quantity of different *var* subsets and Pfsir2 in parasites from children with cerebral malaria either without or with retinopathy (CM-R^**–**^ and CM-R^**+**^). Each dot represents a single isolate. GroupA_median = is the median transcript quantity obtained with primers gpA1 and gpA2 (Table S1). DC8_median is the median transcript quantity obtained with the four-dc8 targeting primers. Group B and C represent the transcript quantity obtained with the primers b1 and c2 (Table S1). The red horizontal bar is the overall median. Significance of difference between CM-R^**–**^ and CM-R^**+**^ are indicated as *p* values calculated using the Mann-whitney U test.

First, as shown in Fig. 1 and Fig. S1, the transcript levels obtained with group A primers including DC13 showed no difference between CM-R^**–**^ and CM-R^**+**^ groups (Fig. 1a,b, Fig. S1a,b). In contrast the primers b1 and c2 targeting general group B and C *var* genes respectively showed higher transcript in the CM-R^**–**^ group (p = 0.002 and p < 0.0001 respectively, Fig. 1d,e). Contrary to our expectations, the median transcript quantity of the four DC8 targeting primers was not significantly different but tended to be higher in the CM-R^**-**^ group (p = 0.05, Fig. 1c). The *var* subset targeted by the primer dc8-4, detected significantly higher levels of transcript in the CM-R^**–**^ group (p = 0.02, Fig. S1f). Pfsir2a which is an enzyme linked to a role in epigenetic silencing/relaxing of *var* gene expression^26^,^27^ was also associated with the CM-R^**–**^ (p = 0.001) but not Pfsir2b (Fig. 1f,g).

The proportion of children with cerebral malaria accompanied by respiratory distress (RD) was higher in the CM-R^**–**^group (Table 1). This raised the possibility that some *var* subsets might be differentially associated with RD explaining the tendency of parasites from the CM-R^**-**^ group to express elevated *var* transcript. If this were the case, we would expect a positive association between the expressions of some *var* subsets and RD. However, in multiple logistic regression analysis, none of the *var* subsets analyzed showed significant positive associations with RD (Fig. S1h) suggesting that the observed negative association between the transcript quantity of some *var* subsets and retinopathy is independent of RD.

**Figure 2:**
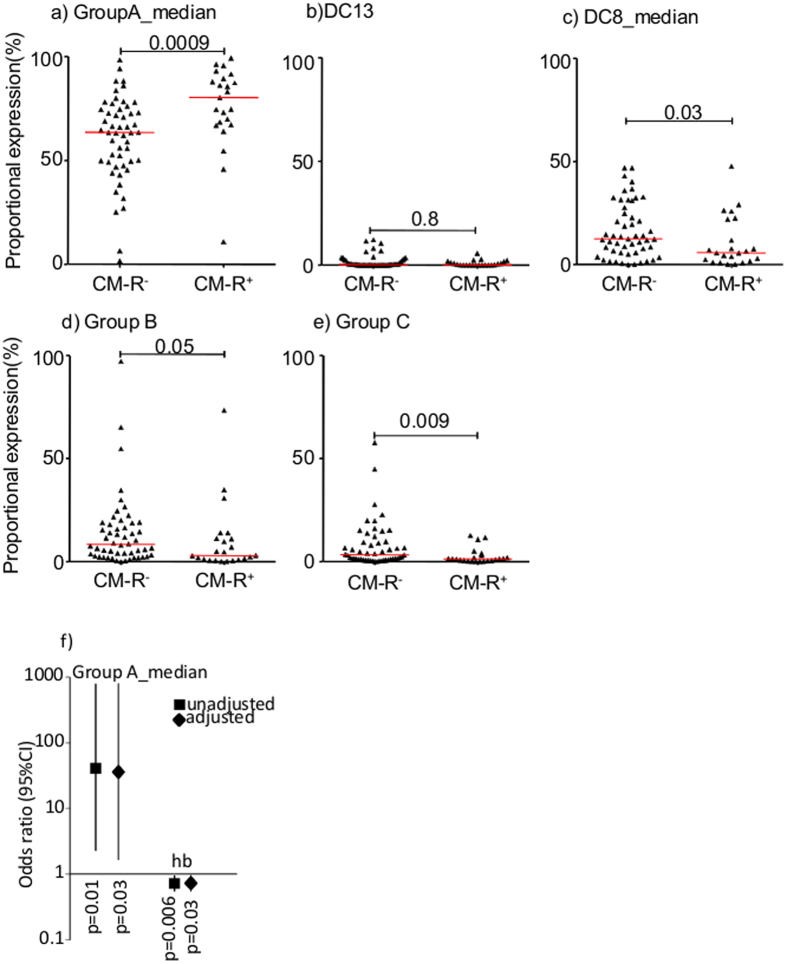
*var* transcript proportional expression and retinopathy. (**a**–**e**) are dot plots showing the proportional expression of broad classes of *var* genes in relation to retinopathy status. Each dot represents a parasite isolate from a child. Group A and DC8 proportional expression represented the proportions of the total measured var transcript contributed by groupA_median and DC8_median. GroupA median and DC8_median are defined in Fig.1 above but calculated in this case as Tu_s_ = 2^(5-∆ct)^. (**f**) Shows a plot of odds ratio and 95%CI of logistic regression models predicting retinopathy. Shown on the left is the association between Group A proportional expression and retinopathy with and without adjusting for admission hemoglobin level. Also shown (on the right) is the association between admission hemoglobin and retinopathy with and without adjusting for group A proportional expression.

The *var* transcript quantity used in the above analysis was calculated relative to the average expression of two reference metabolic genes, and does not provide information on the overall composition of the population of parasites causing infection. We therefore calculated the expression of each of the *var* gene subsets as proportion of total measured *var* transcript and explored the associations of these “proportional expression” values with retinopathy. Of the *var* subsets analyzed, proportional expression of group A *var* genes was positively associated with CM-R^+^ (p = 0.0009, Fig. 2a) while proportional expression of group B and C were negatively associated with CM-R^+^ (p = 0.05 & p = 0.009 respectively, Fig. 2d,e). Contrary to expectation, proportional expression of DC8 var genes was negatively associated with CM-R^+^ (p = 0.03, Fig. 2c) and proportional expression of DC13 var genes showed no difference between the two groups (Fig. 2b).

Proportional expression analysis by qPCR has limitations because different defined subgroups of var genes frequently carry shared sequence features meaning that various primer sets used to quantify var gene expression can amplify overlapping sets of genes. We performed secondary analyses to explore the potential impact of this overlap on our analysis. Previous data suggests that the gpA2 primer tends to amplify some DC8 transcript and dc8-3 primer has limited specificity^19^. In the secondary analysis we therefore used gpA1 primers alone to represent group A and excluded dc8-3 in the calculation of DC8 median transcript. We then re-calculated the proportional expression of the *var* subgroups and tested their associations with retinopathy. Consistent with the primary analysis shown in Fig. 2, proportional expression of group A (gpA1) was positively associated with retinopathy (p = 0.02, Fig. S2a) while group B(b1) and C(c2) were negatively associated with retinopathy (p = 0.03, p = 0.009 respectively, Fig. S2d,e). Proportional expression of DC8 and DC13 showed no difference between the two groups (p = 0.5, p = 0.9 respectively, Fig. S2b-c).

Finally, to estimate proportional expression of non-overlapping *var* groups (A, B and C), we excluded transcript contributed by DC8, DC13 and gpA2 primers from the analysis. With this approach group A (gpA1) proportional expression remains positively associated with CM-R^**+**^ (p = 0.01, Fig. S3a) while group B and C proportional transcript remained negatively associated with CM-R^+^ (p = 0.03, p = 0.01 respectively, Fig. S3b,c).

In previous studies parasites expressing higher proportion of group A-like *var* genes was associated with both clinically defined cerebral malaria and severe malaria anemia^28^. Since children with retinopathy tended to be anemic (Table 1), we used logistic regression to test whether the association between retinopathy and group A proportional expression is explained by the different levels of anemia. We first used each of them as the only explanatory variable in a logistic regression model predicting retinopathy, then in combination to adjust for one another. As shown in Fig. 2f, despite a slight reduction in the odd ratio when adjusted for admission hemoglobin, the group A proportional transcript remained significantly associated with retinopathy (OR(95%CI): unadjusted; (73.97(2.8,1942.0), p = 0.01 adjusted; (42.21(1.41,1267.30), p = 0.03). Similarly admission hemoglobin remained significantly associated with retinopathy when adjusted for group A proportional transcript (OR (95%CI): unadjusted; 0.72(0.56, 0.91), p = 0.006, adjusted; (0.76(0.59,0.97), p = 0.03). This result suggests the association between group A and retinopathy is independent of anemia.

### *var* transcript quantity is associated with Pfsir2 expression

**Figure 3:**
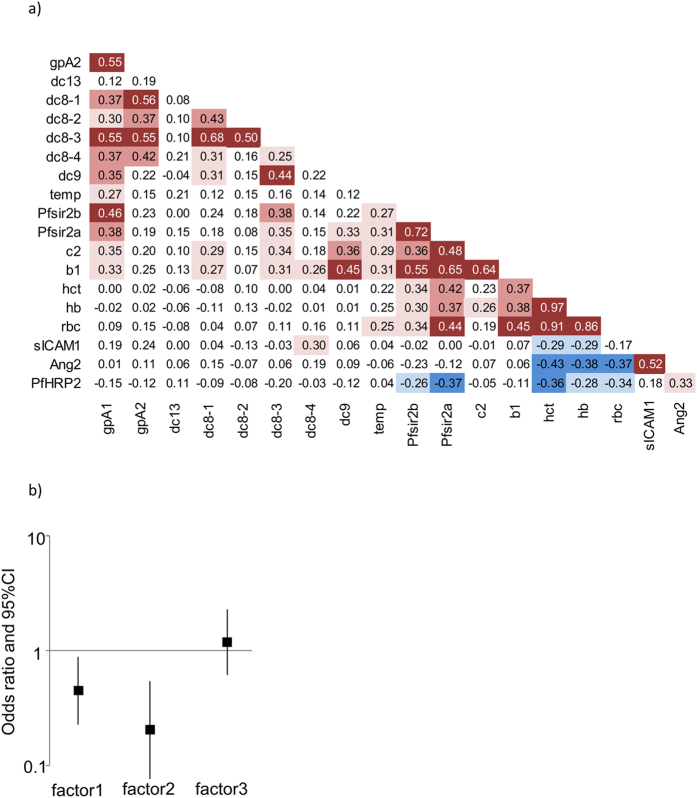
Correlation matrix and Principal factor analysis. (**a**) A correlation matrix: Shown are Spearman’s correlation coefficients of the associations between the variables (N = 62). The background shading is based on the p value of the associations. The darker the background colors the smaller the p value ; dark red = p < 0.0005, mid red = p < 0.005 - ≥ 0.0005, light red = p < 0.05 - ≥ 0.005. mid blue = p < 0.005 - ≥ 0.0005, light blue = p < 0.05 - ≥ 0.005. Red background indicates positive associations and blue background indicate negative associations. (**b**) The relationship between factor scores derived from principal factor analysis and retinopathy: A plot of odds ratio and 95% CI of three logistic regression models predicting retinopathy using either factor 1 factor 2 or factor 3 scores as sole explanatory variables. More details of these factors are shown in Table 2.

A recent work on *P. falciparum* isolates from Gambian Children suggested PfSIR2 involvement in deregulation of *var* gene expression especially those of group B and in the pathogenesis of severe malaria^29^. Surprisingly, there was evidence for elevated PfSIR2 in the retinopathy negative group when compared to the retinopathy positive group. PfSir2a was significantly higher in the retinopathy negative group (Fig. 1f). However, Consistent with Merrick *et al.*^29^. Pfsir2 expression, particularly Pfsir2a, was positively associated with the transcript quantity determined with primers gpA1, dc8-3, b1 and c2 (Fig. 3A) but the strongest association was observed with group B (b1) and C (c2) *var* genes (Fig. 3a: b1; rho = 0.65, p < 0.0001, c2; rho = 0.48, p = 0.0001, Spearman’s rank correlation coefficient). We also observed a positive relationship between Pfsir2 and body temperature (Fig. 3a; p = 0.02 for both, Pfsir2a, and Pfsir2b). However, of these associations, only Pfsir2a association with b1 (adjusted p < 0.0001) and c2 (adjusted p = 0.01) and Pfsir2b with b1 (adjusted p = 0006) remained significant after bonferroni corrections for multiple comparisons.

### Plasma ang-2 associated with PfHRP2

DC8, DC13, and various group A PfEMP1s have been shown to bind vascular endothelial cells through endothelial protein C receptor (EPCR)^12^,^30^. This interaction has been hypothesized to lead to inflammation and endothelial activation^12^,^20^. However, as shown above, despite the proposal that retinopathy acts as a window into broader brain pathology, we did not find evidence for a positive association between retinopathy and DC8 or DC13 expression (Figs 1 and 2). We therefore tested the relationship between a marker of widespread endothelial activation (ang-2) and expression of the various *var* subsets including DC8 and DC13. None of the *var* subsets showed association with ang-2 (Fig. 3a). Instead ang-2 was positively associated with PfHRP2 and sICAM-1 (Fig. 3a). Ang-2 was also negatively associated with admission hematocrit, hemoglobin, and RBC count (Fig. 3a). When bonferroni adjusted for multiple comparisons, only the association between ang-2 and sICAM-1 remained significant (p = 0.002).

### Principal factor analysis

To gain a greater understanding of the pattern of correlation among the variables and to identify clusters of variables that are more closely correlated, we used principal factor analysis. As shown in Table 2, the variables clustered into 3 major factors labeled factor 1, factor 2 and factor 3. Factor 1 explains 55.65% of the variation, and is significantly associated with estimated expression quantity of group A (gpA1, gpA2,) and DC8 (dc8-1, dc8-2, dc8-3, dc8-4). Factor2 that explains 22.57% of the variations is positively associated with estimated expression quantity of group B (b1) and C (c2) *var* genes, Pfsir2a, and Pfsir2b, and axillary temperature. PfHRP2, sICAM-1, ang-2, and 1/hematocrit was positively associated with factor 3, which explains 14% of the variation (Table 2).

To further explore the result of the factor analysis, we used the predicted factor scores as explanatory variables predicting retinopathy. As shown in Fig. 3b, both factor 1 and factor 2 showed negative associations with retinopathy (OR (95%CI): factor 1; 0.45 (0.23, 0.89) p = 0.02, factor 2; 0.20 (0.08, 0.54) p = 0.004) whereas factor 3 showed no association with retinopathy (OR (95%CI) = 1.18 (0.62, 2.27), p = 0.6).

## Discussion

Malarial retinopathy has been shown to be an important clinical marker of *in vivo* sequestration of malaria parasite infected erythrocytes (IE) in the brain^7^. Because the capillaries in the retina are closely connected to those in the brain^31^, the retina provides a window into the pathophysiology underlying cerebral malaria allowing a distinction to be made between children with coma caused by sequestration of parasites and those whose coma has other causes^4^,^7^. As PfEMP1 is thought to be a major ligand expressed on the IE responsible for sequestration, this study aimed to identify PfEMP1 subsets associated with sequestration of IE during retinopathy by comparing the *var* expression profile of parasites from children with cerebral malaria with and without retinopathy (CM-R^+^ and CM-R^–^).

Parasites selected for binding on human brain endothelial cells were recently found to express predominantly a subset of group B and group A PfEMP1 containing domain cassettes 8 and 13 respectively^17^,^18^. These parasites were also found to bind several endothelial cell-lines derived from various organs suggesting this subset of PfEMP1 confer the parasite growth advantage through their strong cytoadhesive properties^17^,^18^,^32^. PfEMP1 containing DC8 and DC13 have been proposed to bind endothelial cells through protein C receptor (EPCR)^12^ leading to a loss of EPCR^20^. Because of the role of EPCR in regulating inflammation, this further led to the hypothesis that parasite interaction with EPCR may induce inflammation, coagulation and endothelial activation^12^,^20^ potentially explaining much of the pathology of cerebral malaria.

Given the ability of retinopathy to distinguish between cerebral malaria caused by parasite mediated brain pathology and other causes with incidental parasitemia^4^, one aim of this study was to determine whether we could identify an association between these specific subsets of PfEMP1 and retinopathy among children with cerebral malaria. However, contrary to our expectation, we found no difference in *var* transcript quantity that would support enrichment in DC8 or DC13 PfEMP1 in children with retinopathy. Overall there was either no difference between the two groups or, higher levels in the retinopathy negative (CM-R^-^) group (Fig. 1 and Fig. S1). The positive associations between Group A proportional transcript and retinopathy suggest IE sequestration in the brain during cerebral malaria may involve group A mediated cytoadhesion. This is consistent with previous studies that showed group A-like *var* genes expression is associated with cerebral malaria^28^,^33^,^34^,^35^. Group A PfEMP1 are generally long molecules with large number of domains^36^. One of the cytoadhesive phenotypes associated with group A PfEMP1 is rosetting^34^,^37^. This involves the adhesion of IE to uninfected erythrocytes. DC13 also carried by a subset of group A genes associated with binding to EPCR via their CIDR domains. However non-DC13 group A *var* genes have also been shown to mediate binding to EPCR^12^. Therefore we cannot exclude the possibility of EPCR binding playing an important role through *var* genes that are not amplified by the primers used to detect DC8 and DC13.

Overall, our result suggests an involvement of group A PfEMP1 in retinopathy. This PfEMP1 subset could potentially support parasite growth through strong cytoadhesive properties consistent with autopsy studies showing that cerebral malaria is associated with parasite sequestration in multiple organs^38^.

We previously examined the association between group A PfEMP1 expression and a marker of widespread endothelial activation ang-2^39^. We found that expression of group A-like *var* genes and plasma ang-2 were independently associated with severe malaria suggesting that group A PfEMP1 do not play a direct role in widespread endothelial activation^39^. We further show here that widespread endothelial activation measured through ang-2 appears not to be the direct result of *var* genes including DC8 and DC13 (Fig. 3a), since there was no evidence for an association. Instead plasma levels of ang-2 were positively associated with PfHRP2 and negatively with admission hemoglobin level (Fig. 3a). The factor analysis further illustrates this result (Table 2). Plasma ang-2, sICAM-1 and PfHRP2 load on the same factor (Table 2). This result is supported by a recent study that showed microvascular obstruction and endothelial activation are independently associated with severe malaria^40^.

It is important to re-emphasise the fact that the estimated quantity of *var* gene transcript expressed, as opposed to the proportional expression, was generally reduced among children with retinopathy. We speculate that this may be the result of congestion. Congestion is defined as excessive accumulation of blood cells in the microcirculation as a consequence of both sequestered IE that reduce the lumen of the blood vessel and increased rigidity of host erythrocytes^31^. Congestion is likely to play an important role in the development of malarial retinopathy. In an adult study, although congestion and sequestration were found to be highly correlated, congestion was a better predictor of coma^41^. Recently Barrera *et al.*^42^ showed that congestion is positively associated with the severity of retinopathy supporting involvement of congestion in malarial retinopathy.

Congestion may influence the overall level of PfEMP1 expressed by the infecting parasite population. Parasite expresses PfEMP1 on the surface of IE to avoid host splenic clearance^43^,^44^,^45^. In previous studies in which the spleen was removed, increased number of mature parasites in peripheral circulation was observed^43^,^44^,^45^ and the circulating population were found to be unable to cytoadhere^43^. As the microcirculation gets congested as a result of cytoadhesion of IE and slowed movement of blood due to increased rigidity of erythrocytes induced by prolonged infection, sequestration of parasites may occur without necessarily expressing high quantity of PfEMP1. In terms of evasion of host antibodies, lowered PfEMP1 expression may give a within-host survival advantage to parasites and might help explain the overall lower *var* gene transcript quantity observed in the parasites from the CM-R^+^ group. However, because we have not directly measured the level of congestion in this study these suggestions are still speculative.

Unlike previous studies, the comparison made in this study is between two groups of children with severe malaria who cannot be distinguished through their clinical symptoms. In previous studies *var* expression comparison was between parasites from children with 1) severe malaria and non-severe malaria^19^,^28^,^46^ or 2) either of the severe malaria syndromes (impaired consciousness, respiratory distress, severe malaria anaemia) and non-severe malaria^19^,^28^, or 3) clinical malaria and asymptomatic infection^47^. In these studies, elevated expression of group A, DC8, or DC13 var genes was observed in severe cases. In a separate manuscript, we have showed that expression of DC8 and DC13 expression was higher in parasites from children with severe malaria compared to non-severe malaria confirming technical consistency with studies conducted elsewhere.

## Conclusions

In summary our data suggests that retinopathy is associated with higher proportional expression of group A *var* genes but overall lower *var* gene transcript quantity. We have suggested that, under the condition of reduced movement of blood in the microcirculation and congestion, parasites may be able to sequester without making the normal level of investment in the expression of PfEMP1.

## Materials and Methods

This study is nested in a published study whose objective was to understand the value of malarial retinopathy in cerebral malaria^21^. Participants included children admitted to Kilifi County Hospital (formally known as Kilifi District Hospital) between July 2005 and December 2011 with a combination of malaria parasites and coma. This means all the children in this study had severe malaria. Retinopathy status was assessed at admission as described previously^21^. In this study we included all those children who were positive for malaria and had a parasite sample frozen in TRIzol available for *var* expression analysis. Plasma PfHRP2 concentration was determined as described in Kariuki *et al.*^21^. Angiopoietin-2 and soluble ICAM-1 (sICAM-1) plasma levels were determined using commercial ELISA kits DANG20 and DY720 respectively from R&D following manufacturer’s protocol.

### Definition of terms

Cerebral malaria was defined as admission with coma (Blantyre coma score≤2) and presence of *P. falciparum* malaria on a Giemsa stained blood-slide without presence of another cause of coma such as hypoglycemia or meningitis^48^. Retinopathy was defined as presence of hemorrhages, peripheral whitening, macular whitening, vessel color changes, and or papilledema^6^. Acidosis was defined as a base excess value of ≤−8.

### *Var* transcript quantification using quantitative PCR

Patient samples were processed as described previously^28^,^34^. A subset of previously described primers (Table S1) was used to quantify *var* genes in qPCR^19^,^46^. These included four primers targeting DC8 (named dc8-1, dc8-2, dc8-3, dc8-4), one primer for each of DC13 (dc13) and DC9 (dc9). Two primers targeting the majority of group A *var* genes (gpA1 and gpA2) were used to quantify group A *var* gene expression (Table S1). In addition, we quantified the expression of group B (b1) and C (c2) *var* genes using primers described in^46^ (Table S1). Expression of two genes; Pfsir2a and Pfsir2b involved in epigenetic control of *var* gene expression was analysed (Table S1). Two housekeeping genes, Seryl tRNA synthetase and Fructose bisphosphate aldolase^49^ were used for relative quantification of the expressed genes. Amplification efficiency of the primers was determined by generation of standard curves over 5 logs (100 ng to 10 pg of IT4 gDNA). All the primers had above 90% amplification efficiency over this range. For the Real-time qPCR, the PCR reaction and cycling conditions were carried out as described in^19^ with the Applied Biosystems 7500 Real-time PCR system. Cycle threshold (Ct) was set at 0.025. Controls with no template were included at the end of each batch of 22 samples per primer and the melt-curves analysed for non-specific amplification. We used genomic DNA from IT4 laboratory parasite line at 10ng/μl as a standard sample included in all plates because we were able to get successful amplification using this line with all the primers used in this study. The ∆∆ct relative quantification method was used to calculate the arbitrary transcript unit (Tu_s_) here referred to as “transcript quantity” using the formula (Tu_s_ = 2^(5-∆∆ct)^) modified from lavstsen *et al.*^19^. When calculating the “proportional expression” of each *var* subset within each sample comparison, we used the formula (Tu_s_ = 2^(5-∆ct)^)^19.^ We assigned a zero Tu value if a reaction did not result in detectable amplification after 40 cycles of amplification, i.e. the Ct value was undetermined. Samples were excluded from the analysis if amplification of either of the two reference genes, i.e. seryl tRNA synthetase and fructose biphosphate aldolase could not be achieved.

### Statistical analysis

As group A and DC8 *var* genes were measured with more than one primer, we used the median transcript quantity (calculated as Tu_s_ = 2^(5-∆∆ct)^) obtained with the various primers targeting group A or DC8 *var* genes to represent group A and DC8 *var* gene expressions, denoted as groupA_median and DC8_median respectively. That is groupA_median = ((gpA1 + gpA2)/2) and DC8_median = (dc8-1 + dc8-2 + dc8-3 + dc8-4-min(dc8-1, dc8-2, dc8-3, dc8-4)-max(dc8-1,dc8-2, dc8-3, dc8-4))/2. Since the primer dc8-3 is less specific^19^, we also recalculated DC8_median after excluding dc8-3 as follows; DC8_median_2 = dc8-1 + dc8-2 + dc8-4-min(dc8-1, dc8-2, dc8-4)-max(dc8-1, dc8-2, dc8-4). To calculate the transcript proportion contributed by each of the *var* subset, we summed the transcript quantity (calculated as Tu_s_ = 2^(5-∆ct)^) of all the *var* subsets analysed, i.e. sum *var* transcript = (groupA_median + dc13 + DC8_median + b1 + c2) or (gpA1 + dc13 + DC8_median_2 + b1 + c2). We then calculated the proportional contribution by each of the subsets. Mann-Whitney U test was used to assess the difference in *var* expression between retinopathy positive and negative groups.

### Logistic regression analysis

To test whether the relationship between proportional expression of group A and retinopathy is independent of anemia, we used three logistic regression models each predicting retinopathy using either 1) group A proportional transcript, or 2) admission hemoglobin, or 3) both as explanatory variables. Malaria retinopathy was a binary variable and was the outcome or dependent variable in all the three logistic regression models. Both group A proportional transcript and admission hemoglobin were normally distributed, and were entered into the logistic regression models as explanatory or independent variables.

#### Correlation matrix

Was generated using Spearman’s rank correlation coefficient test. The exact p value was determined using the command di ‘r(p)’ in Stata.

### Principal factor analysis

In this analysis factors with an Eigenvalue >1 was considered for further analysis. To optimize the factor loadings, we used promax rotation. Loadings >0.3 or < -0.3 were considered significant. We generated predicted factor scores for each individual (using the command “predict” in Stata) and used these generated factor scores as independent variables in a logistic regression analysis with retinopathy as the dependent/outcome variable.

To have the transcript quantity (Tu_s_ values) data normally distributed before use in factor analysis we added 0.1 to all values (to eliminate zeros) and then log transformed all the resultant Tu_s_ values.

All statistical analyses were performed using Stata version 13.

### Ethics statement

Ethical approval was obtained from Kenya Medical Research Institute (KEMRI) Ethical Review Committee (under SSC 1131 and 1249), and written informed consent was obtained from parents/guardians of the study participants. The study methods were carried out in accordance with the approved guidelines.

## Additional Information

**How to cite this article**: Abdi, A. I. *et al.* Differential *Plasmodium falciparum *surface antigen expression among children with Malarial Retinopathy. *Sci. Rep.* **5**, 18034; doi: 10.1038/srep18034 (2015).

## Supplementary Material

Supplementary Information

## Figures and Tables

**Table 1:** Clinical characteristics of the children.

	N	Retinopathy (+)(N = 23)	Retinopathy (–)(N = 52)	*p*
age (months)	72	38 (28–57)	46(31–67)	*0.4§*
Male Sex	72	12/22(54.55)	24/50 (48.0)	*0.6¶*
Respiratory distress	72	2/22 (9.09)	14/50(28)	*0.08¶*
Ax temp (°C)	75	37.8 (37.1–38.8)	38.5 (37.45–39.25)	*0.15§*
Hematocrit (%)	72	17.2(13.3–23.9)	25.6 (22.2–30.9)	*0.003§*
Hb (grams/dl)	72	6(4.5–8)	8.3(7.2–9.8)	*0.003§*
RBC x 106/μl of blood	73	2.6(1.8–3.4)	3.6(3–4.2)	*0.0006§*
Platelet count	75	91(54–140)	117(50–219)	*0.36§*
Acidosis (%)	67	11/19(57.89)	26/48(54.17)	*0.8¶*
PfHRP2	65	28259(13861–34379)	16731 (1984–25467)	*0.03§*
Peripheral Parasitemia	73	128000(12288–588800)	133400(33078–432000)	*0.84§*
Angiopoietin-2 (pg/ml)	73	4326.0(3362.1–5123.8)	3649.6(2736.8–5714.2)	*0.6§*
sICAM-1 (ng/ml)	73	550.3(446.9–648.5)	484.2(407.2–679.4)	*0.6§*
Died (%)	75	6/23(26.09)	9/52(17.31)	*0.4¶*

^Shown are the median and interquartile range or proportions. p-value calculated using either Mann-Whitney U test (§) or chi square test (¶). Acidosis defined as base excess ≤ −0.8^

**Table 2:** Principal factor analysis.

Variables	Factor 1	Factor 2	Factor 3	Uniqueness
gpA1	**0.64**	0.15	0.14	0.43
gpA2	**0.79**	−0.04	0.13	0.35
dc13	0.23	0.06	0.04	0.93
dc8-1	**0.78**	−0.07	−0.01	0.45
dc8-2	**0.68**	−0.14	−0.22	0.63
dc8-3	**0.85**	0.01	−0.23	0.29
dc8-4	**0.36**	0.16	**0.31**	0.66
dc9	0.29	0.29	0.003	0.75
temp	0.13	**0.33**	0.09	0.82
b1	−0.01	**0.80**	0.14	0.36
c2	0.2023	**0.57**	0.064	0.52
Pfsir2a	−0.06	**0.88**	−0.06	0.26
Pfsir2b	0.04	**0.53**	−0.02	0.70
1/hct	0.15	**−0.48**	**0.36**	0.65
PfHRP2	−0.06	−0.24	**0.42**	0.75
sICAM-1	−0.1591	0.17	**0.76**	0.46
Ang-2	0.0561	−0.09	**0.66**	0.54
Eigenvalue	4.52	1.83	1.14	
proportion	55.65%	22.57%	14%	

Principal factor analysis with promax rotation: Three major factors labeled as factor 1, factor 2 and factor 3 were retained on the basis of eigenvalue >1. In bold is the associations (loadings) between the measured variables and the factors (latent variables) that were considered significant (>0.3 or <−0.3). Factor1 represent group A and DC8 var gene subsets and it account for 55.65% of the variation, factor2 represent group B and C var genes, Pfsir2A and Pfsir2B, and it accounts for 22.57% of the variation and factor3 consist of PfHRP2, host hematocrit level, plasma ang-2 and sICAM-1 levels and it accounts for 14% of the variation in the data. 1/hct loads positively on factor3 and negatively on factor2. N = 62. kmo = 0.77.
