# Supplementary material for: Differential Plasmodium falciparum surface antigen expression among children with Malarial Retinopathy
Source: Sci Rep. 2015 Dec 14;5:18034. doi: 10.1038/srep18034 (PMC4677286; doi:10.1038/srep18034)
Supplement: Supplementary Information [file srep18034-s1.pdf]

Supplementary information

Differential *Plasmodium falciparum* surface antigen expression among children with Malarial Retinopathy

Abdirahman I. Abdi[1, 2*], Symon M Kariuki[1,] Michelle K. Muthui[1], Cheryl A. Kivisi[1], Gregory Fegan[1,3], Evelyn Gitau[1,5], Charles R Newton[1,4] and Peter C. Bull[1, 3*]

Figure-S1AA: The relationship between the transcript quantity of each primer and retinopathy

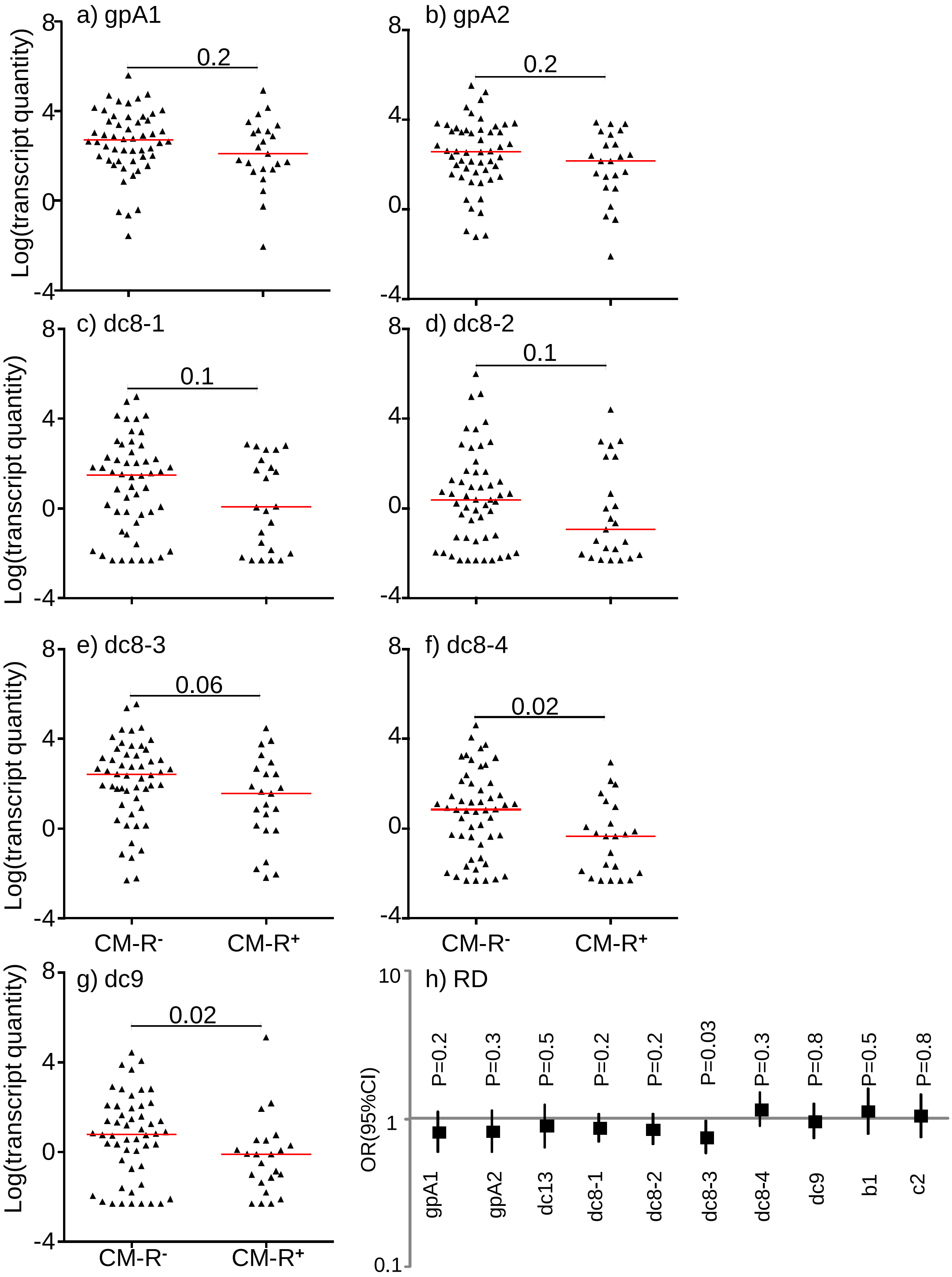

a-g are dotplots showing the relationship between the transcript quantity obtained with the individual primers listed in Table S1 and retinopathy. Each dot represent a parasite isolate. The red horizontal bar is the median. p-value was calculated using Mann-Whitney U test. h) A plot of odds ratio and 95% CI obtained from 10 logistic regression models predicting RD.

Figure-S2AA: Proportional expression of group A, DC8, B, and C

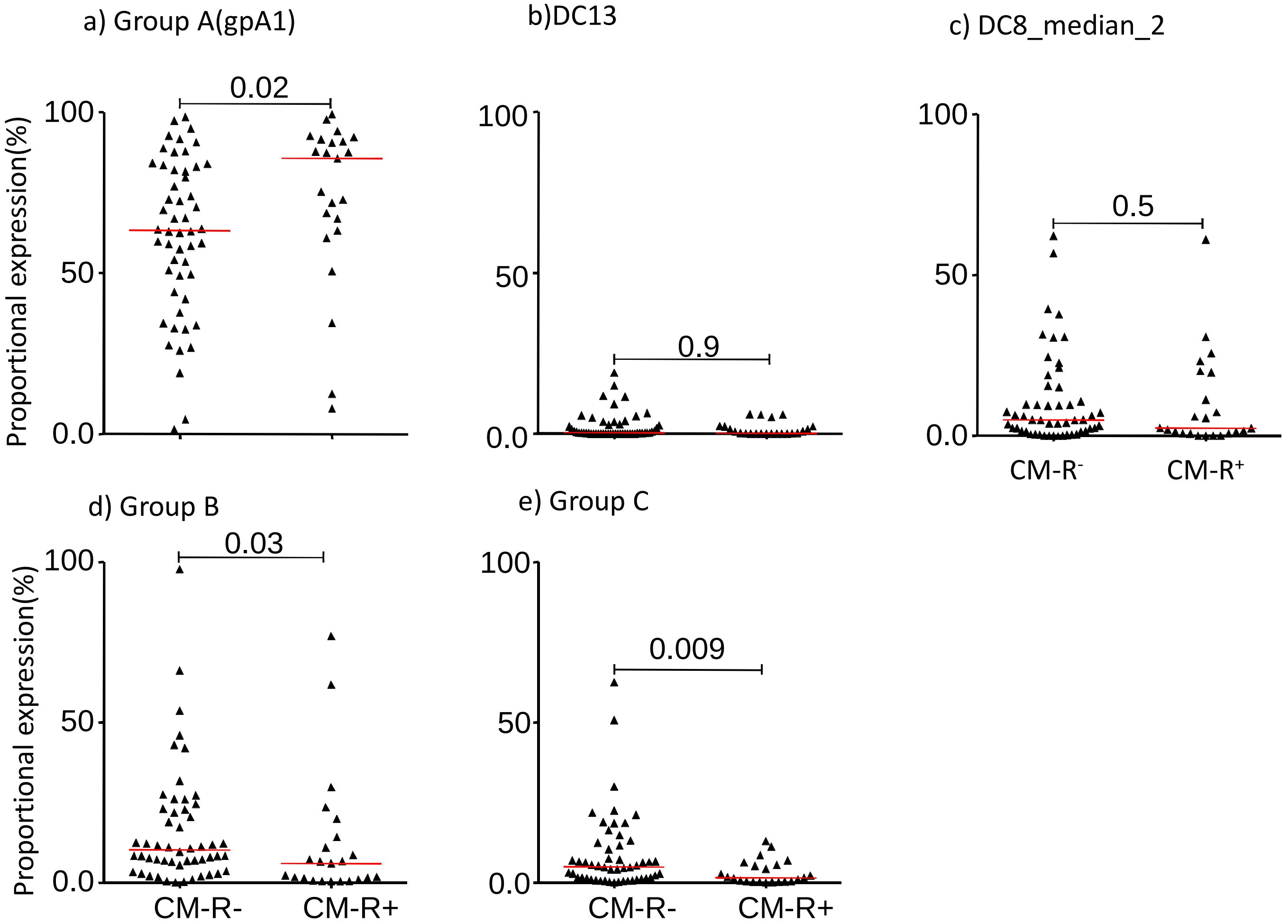

Doplots showing the relationship between the proportion expression of group A, dc8_median group B(b1), group C(c2) *var* genes and retinopathy. Each dot represent a parasite isolate. The red horizontal bar is the median. p-value was calculated using Mann-Whitney U test. In this case dc8-3 transcript was excluded from the calculation of DC8 median transcript (DC8_median_2) and gpA1 only was used to represent group A *var* expression. Total *var* transcript was calculated as follows; sum *var* transcript=-gpA1+ DC8_median_2+ b1+c2. The proportional transcript of the *var* groups was then calculated as described in the text

Figure-S3AA: Proportional expression of group A, B, and C

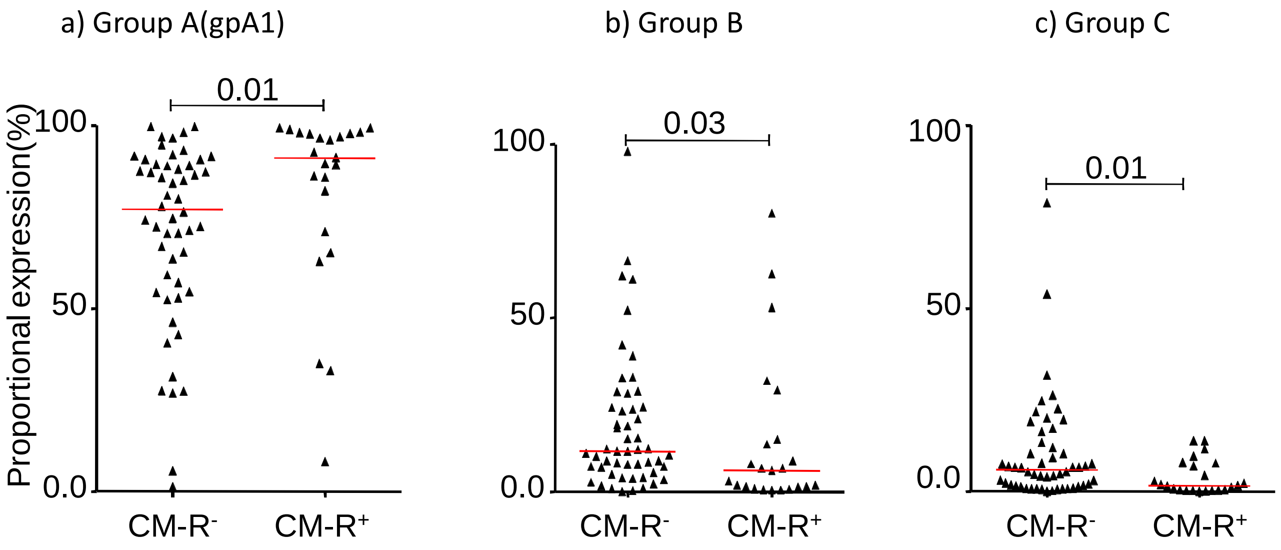

Doplots showing the relationship between the proportion expression of group A(gpA1), group B(b1), group C(c2) *var* genes and retinopathy. Each dot represent a parasite isolate. The red horizontal bar is the median. p-value was calculated using Mann-Whitney U test. Total *var* transcript was calculated as follows; sum *var* transcript=-gpA1+ b1+c2. The proportional transcript of potentially non overlapping *var* groups was then calculated; group A (%)=gpA1 /sum *var* transcript, group B(b1)(%)=b1/sum *var* transcript, group C(c2)(%)=c2/sum *var* transcript .

Table-S1AA: Primers used in this study and their targets

Primer name	Name given	Reference	Target
Dbla_not_var3	gpA1	[1]	Majority of group A *vars*
Dbla2/a1.1/2/4/7	gpA2	[1]	Majority of group A and DC8
CIDR1.4	dc13	[1]	Group A subset containing domain cassette 13
Cidra1.6	dc4	[1]	Group A containing cidra1.6
CIDRa1.1	dc8-1	[1]	Group B subset containing domain cassette 8
DBLa_CIRDa	dc8-2	[1]	Group B subset containing domain cassette 8
DBLb12 & DBLb3&5	dc8-3	[1]	Group B subset containing domain cassette 8
DBLg4/6	dc8-4	[1]	Group B subset containing domain cassette 8
Dblz4	dc9	[1]	Group B containing domain cassette 9
Ups B1	b1	[2]	5' sequence of majority of group B
Ups C2	c2	[2]	5' sequence of majority of group C
Pfsir2a	Pfsir2a	This study	Within coding region
Fwd: CCTTAACAGGGTCAGGTACA,			
Rev: CCAAAAACCCCATATAGTTCCA			
Pfsir2b	Pfsir2b	This study	Within coding region
Fwd:AGGGCCACTAGGTGAAGAAG,			
Rev: GTTGATATGCCAGCACCTGA			
Seryl tRNA synthetase		[3]	
Fructose biphosphate aldolase		[3]	

Primer name is the name of the primer in the original study (see reference column), Name given is the name given to the primer in this study. Primer gpA2 targets group A *var* genes containing dbla1.1/2/4/7 but also could amplify the dbla2 of dc8.

References:

[1] Lavstsen T, Turner L, Saguti F, Magistrado P, Rask TS, Jespersen JS, Wang CW, Berger SS, Baraka V, Marquard AM, Seguin-Orlando A, Willerslev E, Gilbert MT, Lusingu J, Theander TG: Plasmodium falciparum erythrocyte membrane protein 1 domain cassettes 8 and 13 are associated with severe malaria in children. Proc Natl Acad Sci U S A 201[illegible] 109:E1791-800.
[2] Rottmann M, Lavstsen T, Mugasa JP, Kaestli M, Jensen AT, Muller D, Theander T, Beck HP: Differential expression of var gene groups is associated with morbidity caused by Plasmodium falciparum infection in Tanzanian children. Infect Immun 2006, 74:3904-11.
[3] Salanti A, Staalsoe T, Lavstsen T, Jensen AT, Sowa MP, Arnot DE, Hviid L, Theander TG: Selective upregulation of a single distinctly structured var gene in chondroitin sulpha[illegible] A-adhering Plasmodium falciparum involved in pregnancy-associated malaria. Mol Microbiol 2003, 49:179-91.
